# 531. Risk Factors and Therapeutic Interventions Associated with Mortality in Veterans Diagnosed With SARS-CoV-2 Infection (or COVID-19) Admitted to a Large Academic Medical Center

**DOI:** 10.1093/ofid/ofab466.730

**Published:** 2021-12-04

**Authors:** Elwyn Welch, Shazia Raheem, Andrew Hunter, Uma Ayyala, Venkata Bandi, Lavannya Pandit, Christina Kao

**Affiliations:** 1 Michael E. DeBakey Veterans Affairs Medical Center, Houston, Texas; 2 Michael E. DeBakey Veterans Affairs Medical Center/Baylor College of Medicine, Houston, Texas; 3 Michael E DeBakey VA Medical Center, Houston, TX

## Abstract

**Background:**

Patient and treatment-related factors have been used to stratify COVID-19 outcomes; however, studies in the general population and specifically veterans have yielded variable results. This study was designed to assess how baseline characteristics and interventions correlate with clinical outcomes in patients admitted with COVID-19 at a large academic Veterans Affairs hospital.

**Methods:**

Retrospective chart review was conducted on veterans admitted to the hospital with COVID-19 between March 1 to December 31, 2020. Veterans without respiratory symptoms attributed to COVID-19 or enrolled in a COVID-19 clinical trial were excluded. Primary outcome was in-hospital mortality up to 28 days. Secondary outcomes were 90-day mortality, discharge to higher level of care or remained in the hospital within 28 days, and discharge with new oxygen requirement within 28 days. Patient characteristics and therapeutic interventions were assessed for correlation with primary and secondary outcomes.

**Results:**

Of 497 hospitalized patients reviewed, 293 were included for analysis; 94% were male; average age was 68 years with 64.9% of veterans greater than 65 years of age; 43.7% were Black; 17.4% were Hispanic. In-hospital mortality at 28-days and 90-day mortality were 18.1% and 21.5%, respectively. At discharge, 34.1% had a new oxygen requirement and 17.5% went to a higher level of care. Patients that died in-hospital were more likely to be greater than 65 years of age (p< 0.001), Hispanic (p=0.007), have chronic kidney disease (CKD) (p=0.005), be admitted to ICU (p< 0.001); receive dexamethasone (p< 0.001), convalescent plasma (p< 0.001), or antibiotics (p< 0.001); require mechanical ventilation (p< 0.001); or have new onset atrial fibrillation (p< 0.001). Veterans also had higher levels of inflammatory markers within 48 hours of hospital admission (see Table 2) and longer length of hospital stay (< 0.001). There was a trend for patients that died in the hospital within 28-days to be less likely to be Black (p=0.06).

Table 1. Primary and Secondary Outcomes of Study Population (n=293)

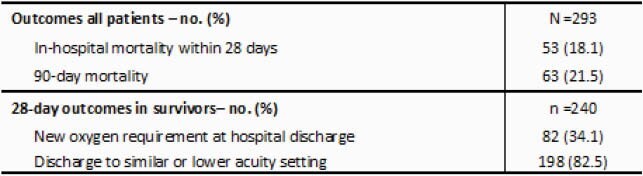

Table 2. Patient Characteristics Stratified by Primary Outcome

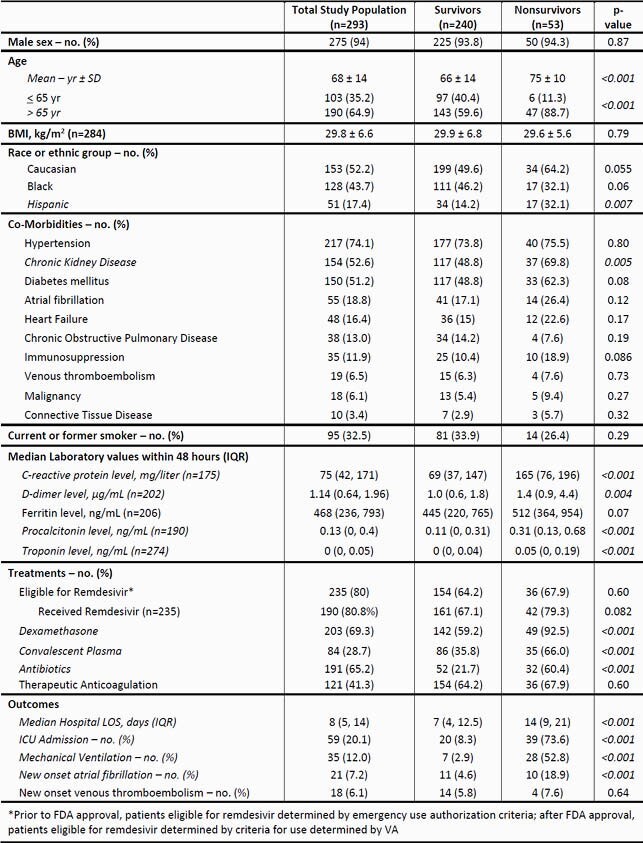

Table 3. Characteristics of the Patients that Survived to Discharge Stratified by Secondary Outcome Measures

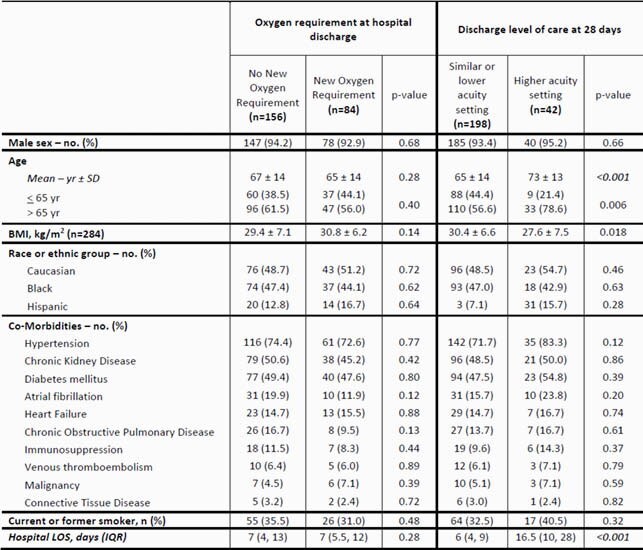

**Conclusion:**

Patients were more likely to die in-hospital within 28-days if they were greater than 65 years of age, Hispanic and had CKD. Veterans that died in-hospital within 28-days had higher inflammatory marker levels and were more likely to receive COVID-19 treatments.

**Disclosures:**

**All Authors**: No reported disclosures

